# Exploring the Antioxidant and Anti-Inflammatory Potential of Saffron (*Crocus sativus*) Tepals Extract within the Circular Bioeconomy

**DOI:** 10.3390/antiox13091082

**Published:** 2024-09-04

**Authors:** Luisa Frusciante, Michela Geminiani, Behnaz Shabab, Tommaso Olmastroni, Giorgia Scavello, Martina Rossi, Pierfrancesco Mastroeni, Collins Nyaberi Nyong’a, Laura Salvini, Stefania Lamponi, Maria Laura Parisi, Adalgisa Sinicropi, Lorenzo Costa, Ottavia Spiga, Alfonso Trezza, Annalisa Santucci

**Affiliations:** 1 Dipartimento di Biotecnologie Chimica e Farmacia, Università di Siena, Via Aldo Moro, 53100 Siena, Italy; luisa.frusciante@unisi.it (L.F.); b.shabab@student.unisi.it (B.S.); tommaso.olmastroni@student.unisi.it (T.O.); scavello2@student.unisi.it (G.S.); martina.rossi892@gmail.com (M.R.); p.mastroeni@student.unisi.it (P.M.); c.nyonga@student.unisi.it (C.N.N.); stefania.lamponi@unisi.it (S.L.); parisi11@unisi.it (M.L.P.); adalgisa.sinicropi@unisi.it (A.S.); lorenzo.costa@unisi.it (L.C.); ottavia.spiga@unisi.it (O.S.); alfonso.trezza2@unisi.it (A.T.); annalisa.santucci@unisi.it (A.S.); 2 SienabioACTIVE, Università di Siena, Via Aldo Moro, 53100 Siena, Italy; 3 Fondazione Toscana Life Sciences, Strada del Petriccio e Belriguardo, 53100 Siena, Italy; l.salvini@toscanalifesciences.org; 4 LifeCARES, Via Emilio Vezzosi 15, 52100 Arezzo, Italy; 5 ARTES 4.0, Viale Rinaldo Piaggio, 34, 56025 Pontedera, Italy

**Keywords:** circular bioeconomy, *Crocus sativus*, *Saccharomyces cerevisiae*, RAW 264.7, antioxidant, anti-inflammatory, MAPK, NF-κB, docking and molecular dynamics simulation, Life Cycle Assessment

## Abstract

Repurposing saffron (*Crocus sativus*) waste presents a sustainable strategy for generating high-value products within the bioeconomy framework. Typically, flower components are discarded after stigma harvest, resulting in significant waste—350 kg of tepals per kilogram of stigmas. This research employed a comprehensive approach, integrating bioactivity studies (in vitro and in silico) with Life Cycle Assessment (LCA) evaluations, to extract and assess bioactive compounds from *C. sativus* tepals sourced in Tuscany, Italy. Phytochemical characterization using UPLC-MS/MS revealed a high abundance and variety of flavonoids in the hydro-ethanolic extract (CST). The antioxidant capacity was validated through various assays, and the ability to mitigate H_2_O_2_-induced oxidative stress and enhance fermentation was demonstrated in *Saccharomyces cerevisiae*. This study reports that *C. sativus* tepals extract reduces oxidative stress and boosts ethanol fermentation in yeast, paving the way for applications in the food and biofuels sectors. Further validation in RAW 264.7 macrophages confirmed CST’s significant anti-inflammatory effects, indicating its potential for pharmaceutical, cosmeceutical, and nutraceutical applications. In silico studies identified potential targets involved in antioxidant and anti-inflammatory processes, shedding light on possible interaction mechanisms with Kaempferol 3-O-sophoroside (KOS-3), the predominant compound in the extract. The integration of LCA studies highlighted the environmental benefits of this approach. Overall, this research underscores the value of using waste-derived extracts through “green” methodologies, offering a model that may provide significant advantages for further evaluations compared to traditional methodologies and supporting the circular bioeconomy.

## 1. Introduction

The concept of the circular bioeconomy is gaining significance across academic, political, and industrial sectors by linking the agendas of the circular economy and bioeconomy to promote sustainability [1]. The European Commission defines the bioeconomy as the use of renewable biological resources from land and sea for energy, industrial, food, and feed production [2], viewing everything, including waste, as a resource. Promoting a circular bioeconomy requires the efficient use of these renewable resources to achieve sustainable solutions through innovative industrial processes that minimize environmental impact.

The circular bioeconomy bridges biotechnology, economy, science, industry, and society, focusing on sustainable economic systems using renewable biological resources. It addresses challenges such as producing resource-efficient products, new biomaterials, and bioenergy [3]. Chemicals for the food industry and therapeutic drug precursors can now be produced directly from waste biomass, offering sustainable alternatives to petrochemical-derived components [4,5,6,7,8,9]. Italy, which adopted a specific bioeconomy strategy in 2017, is a leader in the sector, particularly in bio-based chemistry and compostable bioplastics. Its unique approach is setting a standard in Europe, serving as a model for collaboration among businesses, agriculture, and research [10].

The circular bioeconomy extends product life cycles, reduces waste and emissions, and supports biodiversity and environmental protection [11]. Once a product’s function is fulfilled, its materials can be reintroduced into production, creating additional value. This approach improves environmental quality, soil and marine health, and reintegrates materials and carbon into natural biogeochemical cycles while also increasing employment and revenue in line with the European Green Deal.

The agri-food sector plays a major role in generating vast amounts of by-products. These agricultural waste biomasses hold immense potential for global production across various industries, including bioenergy, animal feed, pharmaceuticals, nutraceuticals, and cosmetics. Biomass encompasses a wide spectrum of biologically derived materials, including firewood, forestry residues, food industry waste, farm effluents, and seaweed, and represents a valuable resource waiting to be tapped [4]. Repurposing them to produce biofuels, generate electricity and heat (biopower), and synthesize chemical compounds (bioproducts) offers a sustainable alternative to technologies reliant on non-renewable fossil-based resources [12,13,14].

Within the framework of a circular bioeconomy, saffron floral residues are a highly valuable resource [15,16,17]. Saffron, one of the most prized spices, has been esteemed since ancient times. This exotic spice is derived from a perennial plant of the genus *Crocus*, belonging to the *Iridaceae* family, botanically known as *C. sativus* L. [18]. Originally utilized by the ancient Egyptians and Romans for its medicinal qualities, saffron later became known primarily as a spice and food coloring [19]. Medicinally used for over 3600 years, saffron was included in opioid preparations for pain relief [20]. Although mainly recognized for its culinary uses, it possesses many health benefits, with its active components showing pharmacological effects such as anticonvulsant, antidepressant, anti-inflammatory, antitumor, and memory improvement [21]. In addition to the renowned “red gold” produced from *C. sativus* stigmas, several by-products from saffron processing are notable for their bioactive compounds [22,23,24]. These by-products are a natural source of antioxidants that can be processed into products for the pharmaceutical, cosmeceutical, and food industries. Moreover, they can be used as fertilizers or enhanced for natural textile dyes, perfume production, and flavored tobacco. The entire flower is edible, characterized by a floral honey aroma, making it suitable for the rising food cultures of veganism and vegetarianism, further promoting sustainable practices [19].

In conventional saffron cultivation, the process of extracting the stigma from the *C. sativus* flower results in the accumulation of anthers, tepals, stamens, and styles as agricultural by-products. These bio-residues are increasingly being considered as valuable as the “red gold” stigma itself. Literature indicates that residues from *C. sativus* processing are rich in antioxidants such as polyphenols, flavonoids, and carotenoids [25,26,27,28,29,30]. These antioxidants can be utilized in foods, beverages, cosmetics, and various pharmaceutical preparations. Among the floral bio-residues of *C. sativus*, the tepals—undifferentiated petals and sepals—are particularly noteworthy for their potential applications. Saffron tepals, which are high in flavonols and anthocyanins, are among the most abundant sources of kaempferol and its glycosides [15,24,31]. Their phytochemical composition endows them with significant antioxidant, anti-inflammatory, and antinociceptive properties [19].

Valued for their minimal side effects and high community acceptance, plant-based medicines are effective for both human and livestock ailments [32,33,34,35,36,37]. The use of natural plant products as alternative therapies is rapidly expanding, with the World Health Organization documenting over 20,000 species of medicinal plants, contributing to about 50% of modern drugs [38]. Key phytochemicals—such as phenolic compounds, saponins, proanthocyanidins, nitrogenous compounds, alkaloids, and terpenoids—exhibit significant pharmacological properties [39,40,41,42,43,44]. Current research is focused on these phytochemicals due to their wide-ranging biological applications, especially in addressing oxidative stress-related conditions [45,46,47,48]. For inflammation treatment, plant-based drugs are being developed as alternatives to non-steroidal anti-inflammatory drugs (NSAIDs), which carry risks of side effects like heart attacks and strokes [33].

Existing studies have highlighted the antioxidant and anti-inflammatory benefits of saffron by-products, yet there is an ongoing and persistent need to delve into the underlying mechanisms of these effects for a thorough understanding. This research addresses this need by employing cell cultures of *S. cerevisiae* and RAW 264.7 macrophages to investigate these mechanisms, conducting in silico analysis to examine interactions, and performing a Life Cycle Assessment (LCA) to evaluate the environmental performances of new potential product value chains. The strength of this approach lies in its holistic view, integrating detailed mechanistic studies with a thorough sustainability assessment. By uncovering the full potential of saffron tepals, we aim to convert them into valuable bioactive compounds within a circular bioeconomy framework, promoting both environmental sustainability and economic value in saffron production.

## 2. Materials and Methods

### 2.1. Materials

Dulbecco’s Modified Eagle’s Medium (DMEM), trypsin-EDTA, and all the reagents used for cell culture were acquired from Merck (Darmstadt, Germany). RAW 264.7 cells were from ATCC (Manassas, VA, USA). The Ames test kit was supplied from Xenometrix (Allschwil, Switzerland).

### 2.2. Preparation of C. sativus Tepals (CST) Extract

The tepals of *C. sativus* were obtained from *La Scoscesa*, a farm located in the Chianti region of Tuscany, Italy. Following harvest, the tepals were thoroughly cleaned and dried at room temperature until a constant weight was achieved. They were then ground and extracted at 80 °C for 3 h using an ethanol–water mixture (70:30 *v*/*v*) with a sample-to-solvent ratio of 1:10 (g/mL). The residual biomass was separated by centrifugation, followed by filtration. The organic solvent was removed through rotary evaporation, and the remaining water was freeze-dried to obtain the dry extract. The extraction process was carried out in duplicate. For subsequent use, 100 mg of the dry extract was dissolved in 1 mL of 100% DMSO to create a 100 mg/mL CST stock solution. Aliquots of this solution were stored at −32 °C for future analysis.

### 2.3. Total Phenolic Content (TPC)

The TPC was measured using the Folin–Ciocalteu (FC) assay [49]. A calibration curve was generated with gallic acid (GA) solutions in the concentration range of 20–120 μg/mL. To prepare the CST samples, the stock solution (1 mg/mL) was diluted with Milli-Q water. Both the standard and sample solutions were mixed with 1 mL of 1N FC reagent in Milli-Q water. After 3 min, 1 mL of saturated Na_2_CO_3_ and 7 mL of Milli-Q water were added to each tube, and the mixtures were incubated for 90 min at room temperature, protected from light. Absorbance was recorded at 725 nm. A blank solution, containing all reagents except for the extract, was also prepared. The TPC is expressed as milligrams of gallic acid equivalent (GAE) per gram of dry extract.

### 2.4. Total Flavonoid Content (TFC)

The TFC was assessed using the aluminum chloride (AlCl_3_) method [50]. A calibration curve was established with quercetin (Q) solutions ranging from 20 to 200 μg/mL. The CST stock solution (1 mg/mL) was diluted with Milli-Q water. In triplicate, 500 μL of the standard or sample was mixed with 100 μL of 10% AlCl_3_ in 1M potassium acetate and 3.3 mL of ethanol. After a 30 min incubation, the absorbance was recorded at 430 nm using a PerkinElmer EnVision system. The TFC results are expressed as milligrams of quercetin equivalent (QE) per gram of extract.

### 2.5. Determination of Reducing Power

The reducing power RP of CST extract was assessed using the potassium ferricyanide reducing power assay, following a modified version of the method described by [51]. A calibration curve was constructed using ascorbic acid (AA) solutions ranging from 20–140 μg/mL. The CST stock solution (1 mg/mL) was diluted with Milli-Q water to concentrations between 25 and 200 µg/mL, with a blank prepared using water.

The samples, standards, and blank were mixed with 1 mL of 0.2 M phosphate buffer (K_2_HPO_4_:KH_2_PO_4_) at pH 6.6 and 1 mL of 1% potassium ferricyanide (K_3_[Fe(CN)_6_]), followed by a 20 min incubation at 50 °C. Afterward, 1 mL of 10% (*w*/*v*) trichloroacetic acid was added to each solution and incubated at room temperature for an additional 10 min. Next, 2.5 mL of Milli-Q water and 0.5 mL of 0.1% (*w*/*v*) ferric chloride (FeCl_3_) solution were added to 2.5 mL of the mixture, and the absorbance was measured at 700 nm. The antioxidant power is expressed as milligrams of ascorbic acid equivalents (AAE) per gram of dry extract.

### 2.6. ABTS Free-Radical Scavenging Activity

The Trolox equivalent antioxidant capacity (TEAC) assay measures the ability of molecules to neutralize oxidized ABTS radicals, converting them back to ABTS [52]. The assay was conducted using the OxiSelect™ TEAC Assay Kit (Cell Biolabs Inc., San Diego, CA, USA), following the manufacturer’s guidelines. In brief, 25 µL of various concentrations of the standard or sample (ranging from 2 to 75 µg/mL) were added to 150 µL of freshly prepared ABTS reagent, diluted 1:50 in the appropriate diluent, in a 96-well plate. After a 5 min incubation on an orbital shaker, the absorbance was measured at 405 nm. The results are expressed as IC50 (µg/mL), representing the concentration required to cause a 50% reduction in absorbance.

### 2.7. DPPH Free-Radical Scavenging Activity

The DPPH free-radical scavenging activity was assessed by measuring the free DPPH (2,2-diphenyl-1-picrylhydrazyl) radical, following the method of Yen and Chen [53], with slight modifications. In summary, 100 µM DPPH was added to each dilution of the standard or sample (ranging from 5 to 100 µg/mL), and the mixtures were incubated in the dark at 37 °C for 30 min. The reaction was monitored at 517 nm to measure the percentage of discoloration. Trolox (T) was used to generate the standard curve. The ability to scavenge the DPPH radical is expressed as IC50 (µg/mL), which represents the concentration needed to achieve a 50% reduction in absorbance.

### 2.8. UPLC-MS-MS

The phytochemical composition of CST was assessed through UPLC-MS-MS analysis using an Ultimate 3000 UPLC system, operated with Thermo Xcalibur software version 4.3.73.11 (Thermo Fisher Scientific, Waltham, MA, USA). The dry CST extract was dissolved in the extraction mixture (ethanol-water 70:30 *v*/*v*) before being injected into the UPLC-Q-Exactive Plus system for analysis, as detailed in a previous study [54].

### 2.9. In Vitro Antioxidant Activity on S. cerevisiae Cell Cultures

#### 2.9.1. Yeast Strain and Culture Conditions

The yeast strain used in this work was *S. cerevisiae* K310, a wildtype wine strain isolated from spontaneously fermenting must. K310 has been extensively studied for its physiological characteristics, protein profile, and stress responses [55,56,57,58,59,60]. K310 was initially cultured in YPD medium at 30 °C with rotary shaking (120 rpm) for 10 h. Following this, a suitable portion of the cell culture was transferred to 150 mL of modified YPD medium (YPDm), adjusted to a pH of 4.5 using 0.2 M citrate/phosphate buffer and containing 100 g/L glucose, to achieve an initial cell density of 1 × 10^4^ cells/mL. The cell suspension was then incubated at 28 °C in the dark without shaking to allow semi-anaerobic growth. CST was introduced to the culture medium at the onset of the exponential growth phase (around the 16th hour, with approximately 3 × 10^6^ cells/mL) to a final concentration of 100 µg/mL.

#### 2.9.2. Growth, Colony-Forming Ability, and Fermentation Assays

Cell growth was monitored by measuring the absorbance of the culture at 660 nm, which helped in selecting the optimal sampling time for subsequent analyses. At 16 h post inoculation, colony-forming ability was assessed in triplicate by plating cell suspensions (ranging from undiluted to 1:100,000 dilutions) on YPD agar. The plates were incubated at 30 °C, and colonies were counted after 2 days. Ethanol levels in the culture medium were measured using an enzymatic assay (kit code 10176290, Boehringer Mannheim, Germany). Briefly, samples from the cell suspensions were quickly cooled and centrifuged (centrifuge 1515R, Eppendorf, Hamburg, Germany). The supernatants were filtered through a 0.2 μm membrane, and ethanol concentrations were determined spectrophotometrically (Agilent 8453 UV-visible spectroscopy system, Waldbronn, Germany) on the filtered samples, which were diluted according to the manufacturer’s instructions [61,62].

#### 2.9.3. Semi-Quantitative Plate SPOT Assay

For this assay, the optical density (OD) at 660 nm of all cell suspensions was measured to determine the volume corresponding to 12 OD for each sample to plate. The corresponding volumes were centrifuged at 3900× *g* for 3 min. After centrifugation, the supernatant was discarded, and the pellet was resuspended in 20 mL of YPD preheated to 30 °C. Each cell suspension was then divided into two 10 mL aliquots. One of the aliquots was treated with 4 mM hydrogen peroxide, and both aliquots were incubated at 30 °C for 30 min. At the end of the incubation, 1 mL of cell suspension was taken from each condition and centrifuged at 12,200× *g* for 2 min. Subsequently, the supernatant was discarded, and the pellet was resuspended in 1 mL of sterile double-distilled water. Next, 200 μL of the cell culture was placed in the first well of a 96-well plate, and 5 serial 1:10 dilutions were performed by adding 180 μL of sterile water and 20 μL of the previous cell suspension. Then, 5 μL of each dilution was spotted onto plates containing solid YPD and incubated at 30 °C for 2 days to allow the growth of K310 and visualization of the results.

### 2.10. In Vitro Anti-Inflammatory Activity on RAW 264.7 Cells

#### 2.10.1. Cell Cultures

RAW 264.7 macrophage cells were purchased from ATCC (Manassas, VA, USA) and cultured in DMEM supplemented with 10% *v*/*v* Fetal Bovine Serum, 100 mg/mL penicillin, and 100 mg/mL streptomycin. The cultures were maintained at 37 °C in a humidified atmosphere with 5% CO_2_. Comparative analyses were conducted using cell populations at the same generation.

#### 2.10.2. Cell Viability

RAW 264.7 cells were seeded at a density of 1 × 10^4^ cells per well in 96-well plates and cultured until they reached 80–85% confluence. The cells were then treated with CST at concentrations of 6, 12, 25, 50, and 100 µg/mL. CST was prepared in dimethyl sulfoxide (DMSO) (Sigma-Aldrich, St. Louis, MO, USA) and diluted in the medium, ensuring that the final DMSO concentration remained below 0.4% *v*/*v* throughout the experiment. A control group was treated with 0.4% *v*/*v* DMSO, matching the highest concentration used in the CST treatments. Cell viability was measured using the Cell Counting Kit-8 (CCK-8) from Sigma-Aldrich (USA), following the manufacturer’s protocol. Absorbance was recorded at 450 nm using a CLARIOstar microplate reader (BMG Labtech, Ortenberg, Germany), and the percentage of viable cells was calculated relative to the vehicle control.

#### 2.10.3. Cell Stimulation

Cells were treated with CST before stimulation with Lipopolysaccharide (LPS) (obtained from *Escherichia coli* O111:B4, Sigma-Aldrich). Dexamethasone (DEX), a standard anti-inflammatory agent, was used as a positive control at a concentration of 5 µg/mL (Sigma-Aldrich).

#### 2.10.4. Quantification of Intracellular ROS Generation

The generation of reactive oxygen species (ROS) in RAW 264.7 cells was assessed using 2′,7′-dichlorodihydrofluorescein diacetate (DCFH_2_-DA). This compound is deacetylated within the cells and subsequently oxidized to the fluorescent 2′,7′-dichlorofluorescein (DCF) [63]. Cells were first pre-treated with CST at concentrations of 25, 50, and 100 µg/mL. Following this, the cells were stimulated with LPS (200 ng/mL) for 5 h. DCFH_2_-DA (10 µM) in Hank’s Balanced Salt Solution was then added to the cells and incubated at 37 °C for 10 min. Fluorescence was measured using an EnVision system (PerkinElmer, Waltham, MA, USA), with an excitation wavelength of 485 nm and an emission wavelength of 535 nm. To determine cell numbers in each well, a Crystal Violet assay was performed [64]. The medium was first removed, and the cells were washed and stained with 0.1% crystal violet at room temperature for 20 min with stirring. After washing, the cells were incubated with 200 µL of pure ethanol for 20 min at room temperature under stirring. Optical density was then measured at 570 nm. The results were normalized to the relative cell count for each well and expressed as the percentage of ROS production compared to the LPS group.

#### 2.10.5. Determination of NO Production

The production of nitric oxide (NO) in the supernatant of RAW 264.7 cells was measured using 6-well plates, where cells were seeded at 1 × 10^6^ cells per well and cultured until they reached sub-confluence (80–85%). Following treatment with CST at concentrations of 25, 50, and 100 μg/mL for 4 h, the cells were stimulated with LPS (200 ng/mL) for 24 h. After stimulation, 100 µL of the conditioned medium from each well was transferred to a new 96-well plate and mixed with an equal volume of Griess reagent, which contained 1% sulfanilamide and 0.1% N-(1-naphthyl) ethylenediamine dihydrochloride in 5% phosphoric acid. The mixture was incubated at room temperature for 10 min. Absorbance was then measured at 540 nm using an EnVision system (PerkinElmer). The nitrite concentration was determined by comparing the results to a sodium nitrite standard curve.

#### 2.10.6. Protein Extraction

Whole-cell lysates were prepared using RIPA buffer supplemented with phosphate and protease inhibitors. The cell lysates were then sonicated for 15 min in an ice bath to facilitate disruption. Protein concentration was determined using a bicinchoninic acid protein (BCA) assay. For nuclear fractionation, NE-PER™ Cytoplasmic and Nuclear Protein Extraction Kit (Thermo Fisher Scientific, Rockford, IL, USA) was used following the manufacturer’s instructions.

#### 2.10.7. Western Blotting

First, 20 μg of protein were resolved by SDS–PAGE and transferred onto a nitrocellulose membrane. The membrane was blocked in TBS 5% *w*/*v* nonfat dry milk at RT with gentle shaking for 2 h. The membrane was incubated with anti-iNOS (rabbit polyclonal IgG, 1:10,000 Sigma-Aldrich), anti-COX-2 (rabbit polyclonal IgG, 1:4000 Sigma-Aldrich), anti-NF-κB p65 (mouse monoclonal clone 1G10.2, 1:1000 Sigma-Aldrich), anti-Nucleolin (rabbit polyclonal, 1:10,000 Sigma-Aldrich), anti-pJNK and anti-JNK (rabbit polyclonals, 1:1000 Sigma-Aldrich), anti-pp38 (1:1000 Sigma-Aldrich), anti-p38 (1:10,000 Sigma Aldrich), anti-ERK (1:20,000 Sigma-Aldrich), and p-ERK (1:2000 Cell Signaling), and anti-GAPDH HRP-conjugated (1:50,000 Sigma Aldrich) primary antibodies, ON, at 4 °C. The blots were washed three times and incubated with anti-rabbit HRP-conjugated secondary antibody (Sigma-Aldrich) 1:80,000 or anti-mouse HRP-conjugated secondary antibody (Sigma-Aldrich) 1:50,000 for 1 h, RT. After washing three times, immunoreactive bands were detected using ECL (LuminataCrescendo, Merck Millipore, Burlington, MA, USA) and images acquired by LAS4000 (GE Healthcare, Chicago, IL, USA). The optical densities of immunoreactive bands were analyzed by ImageQuantTL software (GE Healthcare, Chicago, IL, USA, V 7.0) using GAPDH, Nucleolin or JNK as loading normalizing factors.

#### 2.10.8. Immunofluorescence Study

RAW 264.7 cells were grown on glass coverslips for 24 h. They were pre-treated with CST at 100 μg/mL for 4 h, then stimulated with LPS for 1 h. Following stimulation, the cells were fixed with 4% paraformaldehyde in PBS for 15 min and then washed three times. To permeabilize the cells, they were exposed to 0.5% Triton X-100 in PBS for 5 min. The cells were then incubated with 5% Normal Goat Serum (NGS) in PBS for 20 min, followed by incubation with a 1:200 dilution of anti-NF-κB p65 mouse monoclonal antibody (clone 1G10.2) (Sigma-Aldrich) overnight at 4 °C. After three washes with PBS, the cells were stained with a 1:100 dilution of Alexa 594-conjugated goat anti-mouse IgG (Life Technologies, Carlsbad, CA, USA) for 1 h in the dark at room temperature. Following three additional washes with PBS and one wash with distilled water, the cells were mounted with FluoroShield containing 4′,6-Diamidino-2-Phenylindole (DAPI). Fluorescence images were captured using a Zeiss AxioLabA1 microscope (Oberkochen, Germany).

### 2.11. In Silico Studies

#### Structural Resources and Docking Simulation

The potential targets involved in the interaction with KOS-3 were identified using Swiss Target Prediction [65] using as input for the target research the “canonical SMILES” of Kaempferol 3-O-sophoroside (Compound CID: 5282155), retrieved on PubChem database [66]. Swiss Target Prediction provided six different targets (Table 1) potentially involved in the interaction with KOS-3, and all targets with a “Probability” score of at least 35% were selected; thus, their 3D structures were downloaded through RCSB PDB [67].

The primary structure of the Aldose reductase (AR) homolog protein of the *S. cerevisiae* (target sequence) was retrieved from the UniParc database with entry “UPI00226304FE” [68]. To optimize the 3D structures of each target for the docking simulation, the potential missing side chains and steric clashes in 3D structures reported in PDB files were added/resolved with molecular/homology modelling using MODELLER v.9.3 implemented in PyMOD3.0 (PyMOL2.5 plugin) [69]. Then, 3D structures were analyzed and validated with PROCHECK v.3.5.4 [70]. To reinforce the reliability of our simulations, we applied a docking simulation based on in vitro evidence. Thus, we only selected targets whose experimental 3D structures were combined with an active compound, which was used as a reference to create a box enclosing all binding residues. The docking simulation was performed using Autodock/VinaXB implemented in the PyMOL2.5 plugin, and it was set with an exhaustiveness of 32, and all other parameters by default [71,72]. MGLTOOLS v.1.5.7 scripts and OpenBabel v.3.1.0 were used to respectively convert protein and ligand files and to add Gasteiger partial charges [73,74]. The interaction network was explored with the P.L.I.P. v. 2.3.0 Tool [75,76].

### 2.12. Mutagenicity Assay: Ames Test

The TA100 and TA98 strains of *Salmonella typhimurium* were utilized for mutagenicity assay in the absence and presence of metabolic activation, i.e., with and without S9 liver fraction. The tester strains used were selected because they are sensitive and detect a large proportion of known bacterial mutagens and are most commonly used routinely within the pharmaceutical industry [77]. The following specific positive controls were used, respectively, with and without S9 fraction: 2-Nitrofluorene (2-NF) 2 µg/mL + 4-Nitroquinoline N-oxide (4-NQO) 0.1 µg/mL, and 2-aminoanthracene (2-AA) 5 µg/mL. The final concentration of S9 in the culture was 4.5%.

Approximately 107 bacteria were exposed to 6 concentrations (0.025, 0.050, 0.10, 0.50, 1.0, and 10.0 mg/mL) of the CST extract, as well as to positive and negative controls, as described before [78].

### 2.13. Life Cycle Assesment (LCA)

Calculations were performed implementing a cradle-to-gate approach using primary data coming from experiments [79,80] (see Figure S1 in the Supplementary Material). The software SimaPro version 9.3.0.3 was used in combination with the Ecoinvent database version 3.7.1 [81] for secondary data. The environmental impact assessment method selected was the EF 3.0 Method (adapted) v1.01 [82] that allows for the characterization of 16 environmental indicators.

### 2.14. Statistical Analysis

Experiments were performed in triplicate. Statistical analyses were performed with GraphPad Prism 9.0 software (GraphPad Software, San Diego, CA, USA). Data are presented as mean ± SD and were compared using the unpaired *t*-test or the one-way ANOVA with an appropriate post hoc test. A *p* value of 0.05 or less was considered significant.

## 3. Results

### 3.1. Chemical Composition and Antioxidant Capacity of CST

The *C. sativus* tepals extract was obtained by heat-reflux extraction. This process used 10 g of oven-dried, pulverized *C. sativus* tepals sourced from the Tuscany area of Chianti, with an ethanol–water mixture (70:30 *v*/*v*) as the solvent. The extraction produced 4.439 g of freeze-dried CST, resulting in a percentage yield of 44.39 ± 1.93 (*w*/*w*). to evaluate the extraction efficiency. The antioxidant ability of the extract was subsequently assessed by measuring its reducing power and radical scavenging activity. Table 2 presents the calculated values for TPC (mg GAE/g dry extract), TFC (mg QE/g dry extract), RP (mg AAE/g dry extract), and radical scavenging activity (IC50 µg/mL) of CST.

CTS revealed calculated TPC and TFC values of 80.05 ± 5.11 mg GAE/g and 38.36 ± 1.22 mg QE/g, respectively, as shown in Table 2. These results were associated with a significant antioxidant capacity (Table 2), demonstrating a stronger effect on the ABTS radical compared to DPPH, as indicated by the IC50 values (µg/mL), which represent the concentration needed to achieve a 50% reduction in absorbance.

CST extract was further analyzed using UPLC-MS/MS; metabolites were identified through Compound Discoverer 3.3 software integrated with the ChemSpider database and mzCloud. Table 3 reports the most representative metabolites (>0.1% peak area %) found in CST, along with their retention time, molecular formulae, calculated MW, *m*/*z*, and error (ppm). Compounds with an area < 0.1% were considered traces (Tr.) and are reported in Table S1. For all matched compounds, the error was lower than 5 ppm.

### 3.2. CST Reduced H_2_O_2_-Induced Oxidative Stress in S. cerevisiae K310

**Figure 1:**
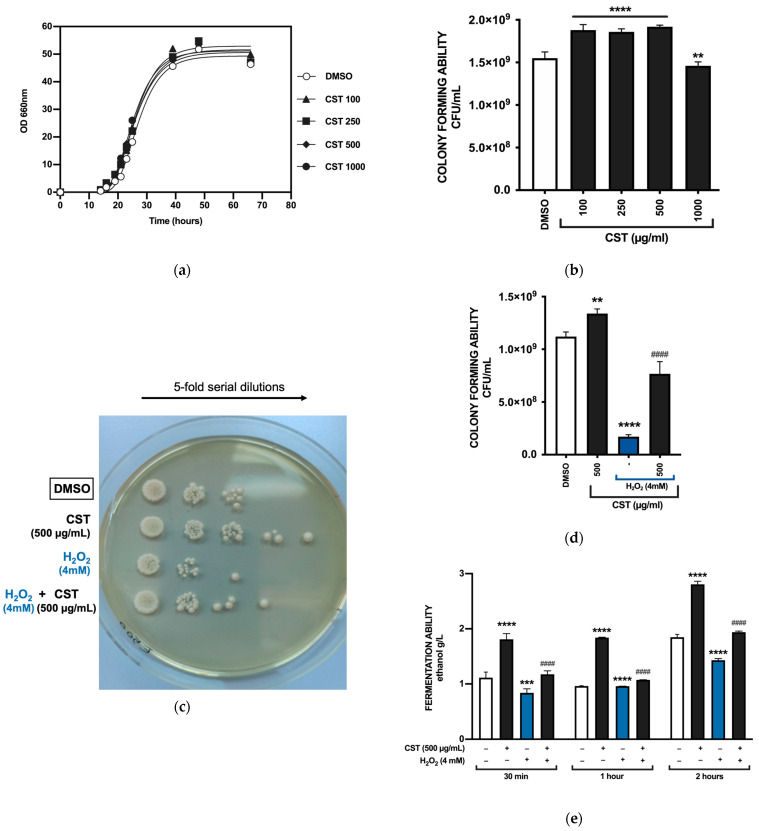
Effect of CST on the parameters of *S. cerevisiae* K310 cells. (**a**) Growth of K310 cultured in YPDm medium (glucose, 100 g/L; pH 4.5; incubated at 28 °C under semi-aerobic conditions) with CST (100, 250, 500, and 1000 µg/mL). (**b**) Colony-forming ability of K310, expressed as CFU/mL, upon treatment with concentrations of CST for two days. Experiments were performed in triplicate. Results are expressed as CFU/mL and compared using one-way ANOVA with Dunnet’s post hoc test. ** *p* = 0.0026; **** *p* < 0.0001 (vs. DMSO) (**c**) Serial dilutions of K310 cells and (**d**) colony-forming ability of K310 cells treated with 500 μg/mL of CST in the presence or absence of 4 mM H_2_O_2_. Colonies were counted after two days of incubation at 28 °C on YPD plates. Data are reported as CFU/mL. Results are expressed as mean ± SD and compared using one-way ANOVA with Tukey’s post hoc test. ** *p* = 0.0017; **** *p* < 0.0001 (vs. DMSO); #### *p* < 0.0001 (vs. H_2_O_2_). (**e**) Fermentation ability of K310 cells, treated or untreated with 4 mM H_2_O_2_ after 16 h of inoculation, evaluated in the presence or absence of CST (500 μg/mL). Cells were treated for 30 min, 1 h, and 2 h with H_2_O_2_. Data are reported as ethanol g/L. Results are expressed as mean ± SD and compared using one-way ANOVA with Tukey’s post hoc test. *** *p* = 0.0004; **** *p* < 0.0001 (vs. DMSO); #### *p* < 0.0001 (vs. H_2_O_2_).

The potential in vitro antioxidant effect of CST was first evaluated using a yeast cell model obtained by treating *S. cerevisiae* K310 cells with 4 mM H_2_O_2_. Figure 1a displays the growth kinetics of K310 under control conditions (YPD medium supplemented with DMSO) and in the presence of CST at concentrations of 100, 250, 500, and 1000 µg/mL. The extract was added at the onset of exponential growth (16 h), a time when the cells were most susceptible to stress, and growth was subsequently monitored. Notably, CST did not affect the growth rate of K310 at any of the tested concentrations, and growth resumed normally. The control was performed by treating the cells with DMSO, serving as the vehicle at the same concentration as the highest CST dose. DMSO concentration remained below 1% (*v*/*v*) and did not negatively impact the analyzed parameters. According to the growth curve, the K310 strain was in the early exponential phase at 16 h and transitioned to the early stationary phase by 42 h (Figure 1a). Cell viability assays were conducted at 16 h to assess cell survival following CST administration, allowing for the identification of optimal concentrations that could enhance antioxidant activity (Figure 1b). A concentration of 500 µg/mL was selected as the optimal CST concentration for further analysis.

A SPOT viability assay was conducted to assess the response of yeast cells to oxidative stress induced by H_2_O_2_, both with and without CST (Figure 1c). This visual assay qualitatively indicates yeast cell viability by showing cell density in single spots on an agar plate. Cells untreated or treated with CST at 500 μg/mL were exposed to 4 mM H_2_O_2_ before plating. A drop of the undiluted K310 culture (density of 4 × 10^5^ cells/mL) and five subsequent serial dilutions were plated on agar to compare cell viability. After 2 days of incubation at 28 °C, images were captured. The results showed differences in cell growth across various treatments, with the addition of CST improving cell viability up to the fifth dilution, while the control group exhibited viability only up to the third dilution. In the presence of H_2_O_2_, the trend was similar, with CST increasing the number of colonies in each spot.

We further assessed the colony-forming and fermentative abilities of yeast cells under oxidative stress conditions. Figure 1d illustrates how CST influences the colony-forming ability of the cells. This viability assay involved inoculating the culture, performing serial dilutions up to 10^−5^, and plating 100 μL of the final suspension on YPD agar to evaluate cell counts on each plate. As seen with SPOT assay, a concentration of 500 μg/mL CST was used, and the plates were incubated at 28 °C for 2 days. Our results confirmed that CST not only did not harm cell viability but significantly enhanced it. While H_2_O_2_ at a concentration of 4 mM notably reduced the number of CFU/mL, our data indicated an increase in viability in samples treated with CST, regardless of the oxidative stress condition (Figure 1d).

H_2_O_2_ negatively impacted the fermentative ability of yeast (Figure 1e). The ethanol assay measures the amount of ethanol produced by cell cultures at different growth stages. In the presence of alcohol dehydrogenase, ethanol is oxidized to acetaldehyde by nicotinamide adenine dinucleotide (NAD). To assess the effect of CST on fermentation, we calculated the ethanol levels in the culture medium under standard conditions and after oxidative stress induction with H_2_O_2_. Figure 1e illustrates the fermentative capability of *S. cerevisiae* K310 cells grown in YPD medium, both with and without CST at a concentration of 500 μg/mL, and with or without H_2_O_2_ (4 mM) after 30 min, 1 h, and 2 h. The results indicate that the fermentative capacity of yeast cells treated with 500 µg/mL CST is greater than that of the control. These findings suggest an increased fermentative capacity, indicating that CST positively influences the fermentative ability of *S. cerevisiae* even under oxidative stress conditions.

#### In Silico Studies

**Figure 2:**
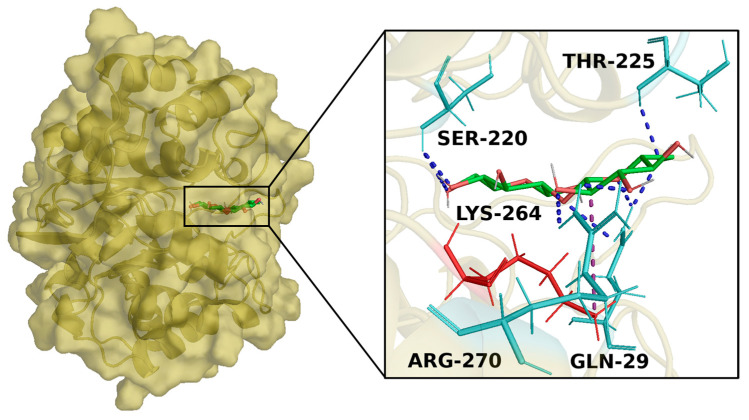
Overview of yeast AR docked with KOS-3. The target 3D structure is depicted in a yellow surface and cartoon, while KOS-3 is reported in green (carbon atom), red (oxygen atom), and white (hydrogen atom) sticks. Enlarged view of the docked pose of KOS-3 within the target binding pocket. In cyan and red sticks are represented the binding residue forming h-bonds (blue dotted line) and salt bridge (purple dotted line), respectively.

Swiss target prediction provided five different human targets potentially involved in the interaction with KOS-3. To identify the potential yeast targets, a Multiple Sequence Alignment was performed using BLASTP 2.16.0 tool between the primary structure of each target predicted against the “*Saccharomyces cerevisiae*” database. From MSA results, only one target showed the homolog protein in the yeast, that is, the human AR, showing identity and coverage of 44.03% and 90%, respectively. The 3D structure obtained with Modeller implemented in PyMOD3.0 (using as template sequence the human AR with PDB code 1Z8A) showed a RMSD of 0.260 Å following a structural superposition between the template (human) and target (yeast) 3D structures, exhibiting a good quality of the yeast target 3D structure. Thus, a docking simulation between KOS-3 and the yeast target was performed allowing the compound to bind in a known binding region of human AR inhibitors. Docking results showed a binding affinity of -5.5 kcal/mol, revealing the ability of the compound to spontaneously bind against the target. Interaction analyses detected a wide polar interaction network; in detail, KOS-3 trigged h-bonds with Gln-29, Ser-221 (human Ser-210), Lys-264 (human Lys-262), and Arg-270 (human Arg-268), and a salt bridge with Lys-264 (Figure 2). Pairwise sequence alignment showed that KOS-3 was able to form the same strong polar interactions with sensing residues of the human target in complex with the known inhibitor of human AR [83].

### 3.3. CST Reduced LPS-Induced Inflammation in RAW 264.7 Cells

**Figure 3:**
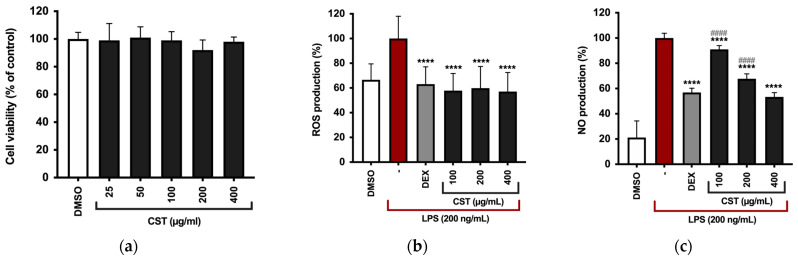

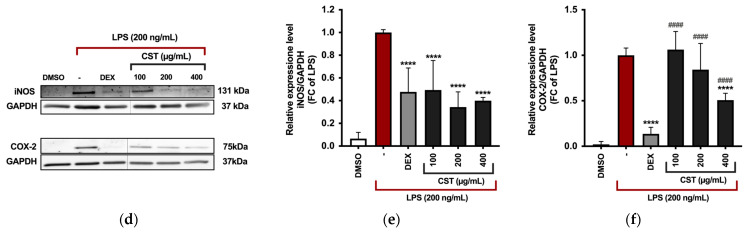
(**a**) After culturing RAW 264.7 cells with CST (0, 25, 50, 100, 200, and 400 μg/mL), cell viability was evaluated using the CCK-8 assay after 24 h. (**b**) Intracellular ROS levels were measured upon pre-treatment with concentrations of CST, and stimulation (LPS 200 ng/mL) for 5 h. Results are shown as bar graphs illustrating ROS levels measured by relative fluorescence intensity normalized to cell count using the Crystal Violet assay. **** *p* < 0.0001 (vs LPS). *p*-values were calculated by one-way ANOVA with Tukey’s post hoc test. (**c**) Effect of CST on LPS-induced inflammatory in RAW264.7 cells. RAW 264.7 cells were first pre-treated with DEX or CST for 4 h, followed by stimulation with 200 ng/mL LPS for 24 h. The figures depict (**c**) NO production evaluated by Griess assay, (**d**,**e**) iNOS, and (**d**,**f**) COX-2 expression determined using Western blotting. Immunoreactive bands were analyzed using GAPDH as a loading normalizing factor. Spliced sections of the Western blot images, which are delineated by a black line, originate from the same original image. All data are presented as mean ± SD of three independent experiments. Statistically significant differences were indicated by **** *p* < 0.0001 (vs. LPS); #### *p* < 0.0001 (vs. DEX). *p*-values were calculated using one-way ANOVA with Tukey’s post hoc test.

The RAW 264.7 murine macrophage cell line was employed to evaluate the anti-inflammatory potential of CST. Initially, the extract’s potential cytotoxic effects were assessed using the CCK-8 assay. Results in Figure 3a depict cell viability as a percentage compared to the DMSO-treated control, where DMSO served as a vehicle; its final concentration did not exceed 0.4% (*v*/*v*) in either treated or untreated cells and did not adversely affect the analyzed parameters. None of the tested concentrations of CST had an impact on RAW 264.7 cell viability (Figure 3a).

Subsequently, to evaluate the extract’s ability to mitigate the production of ROS, RAW 264.7 cells were pre-treated with CST at concentrations of 100, 200, and 400 µg/mL, and then stimulated with LPS (200 ng/mL) for 5 h. Dexamethasone (DEX) at 5 µg/mL was used as a positive control. ROS levels were quantified by DCFH_2_-DA and normalized to cell number with Crystal Violet staining. As shown in Figure 3b, all tested concentrations significantly reduced ROS production in LPS-stimulated RAW 264.7 cells.

To measure the impact of CST on the production of key inflammatory mediators in RAW 264.7, the cells were pre-treated with CST at concentrations of 100, 200, and 400 µg/mL or DEX at 5 µg/mL for 4 h, followed by stimulation with 200 ng/mL LPS for 24 h. NO release was assessed using the Griess assay. DEX significantly inhibited NO production in the supernatant of RAW 264.7 cells (Figure 3c), and CST also significantly reduced NO production at all tested concentrations.

Western blotting was employed to evaluate the protein expression of iNOS, the enzyme responsible for NO production, and COX-2. Figure 3d–f illustrate a dose-dependent reduction in the expression of these enzymes compared to the LPS-stimulated group. Quantitative analysis of the immunoreactive bands indicated decreased iNOS expression in RAW 264.7 cells treated with CST at 100, 200, and 400 μg/mL, which correlates with the NO assay findings. COX-2 expression significantly decreased at the highest concentration of the extract (Figure 3f), thus confirming the extract’s anti-inflammatory effect.

**Figure 4:**
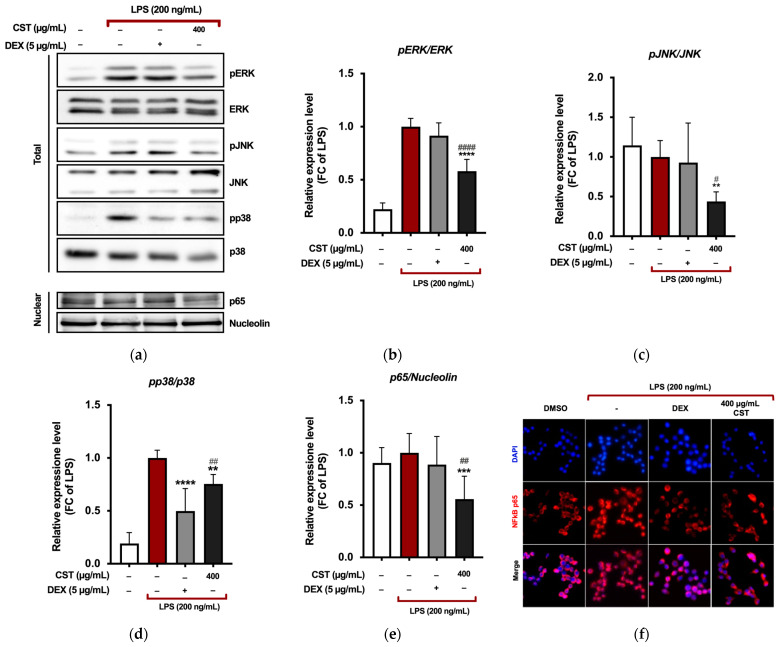
(**a**) The MAPK and NF-κB signaling pathways were impacted by treatment with CST in LPS-stimulated RAW 264.7. Cells were pre-treated with 400 µg/mL of CST for 4 h and subsequently stimulated with LPS (200 ng/mL) for 1 h. The effect of CST on the phosphorylation of (**b**) pERK, (**c**) pJNK, and (**d**) p38 and (**e**) the nuclear expression of the NF-κB p65 subunit was investigated by western blot analysis. Relative band intensities were quantified from three independent experiments using densitometry. Data are presented as mean ± SD of three independent experiments. ** *p* ≤ 0.0044, *** *p* = 0.0004, and **** *p* < 0.0001 (vs. LPS); # *p* = 0.0144, ## *p* ≤ 0.0097, and #### *p* < 0.0001 (vs. DEX). *p*-values were calculated using one-way ANOVA with Tukey’s post hoc test. (**f**) Visualization of NF-κB localization was performed using fluorescence microscopy after staining for NF-κB p65 (red). Cell nuclei were counterstained with DAPI (blue). Images were captured at 40× magnification.

To investigate the mechanisms involved in the inflammatory response, we analyzed the phosphorylation of proteins in the mitogen-activated protein kinase (MAPK) pathway and the nuclear expression of the nuclear factor kappa-light-chain-enhancer of activated B cells (NF-κB) p65 subunit in LPS-activated RAW 264.7 cells using Western blotting (Figure 4a). LPS stimulation increased the phosphorylation of ERK, which was suppressed by CST treatment (Figure 4b). The results also demonstrated that CST significantly reduced the phosphorylation of JNK and p38 (Figure 4c,d).

The NF-κB transcription factor is crucial in mediating the production of the observed inflammatory markers by directly regulating the expression of genes encoding enzymes such as iNOS and COX-2 [84]. Therefore, we further investigated the effects of CST by assessing the expression of the p65 subunit in the nucleus of RAW 264.7 cells following LPS induction. Treatment with CST (400 µg/mL) significantly reduced the expression of NF-κB p65 in the nucleus of LPS-stimulated RAW cells (Figure 4e). Immunostaining and fluorescence microscopy analysis of NF-κB p65 localization revealed that upon LPS stimulation, the p65 protein was predominantly localized in the nucleus of RAW 264.7 cells (Figure 4f). However, CST at a concentration of 400 µg/mL prevented the nuclear translocation of NF-κB, retaining it in the cytoplasm.

These data indicate that CST inhibits the LPS-stimulated inflammatory signaling by suppressing the phosphorylation of MAPK proteins and preventing the nuclear translocation of NF-κB p65.

#### In Silico Studies

Swiss target prediction provided five different human targets potentially involved in the interaction with KOS-3, the most representative compound found in CST. A docking simulation between KOS-3 and the targets was performed, allowing the compound to bind in their sensing regions. Docking results showed that KOS-3 was able to spontaneously bind against all targets, exhibiting a binding affinity of −8.1 kcal/mol for Neuromedin-U receptor 2, −7.8 kcal/mol for Alpha-2a adrenergic receptor, −8.4 kcal/mol for adrenergic receptor Alpha-2, −3.9 kcal/mol for Acetylcholinesterase, and −5.5 kcal/mol for AR. Thus, the adrenergic receptor Alpha-2/KOS-3 complex was selected as the best complex for further in silico analyses.

Pairwise sequence alignment showed a high homology between human and mouse ADRA2C, exhibiting a cover and identity of 97% and 92.04%, respectively (Figure S2).

**Figure 5:**
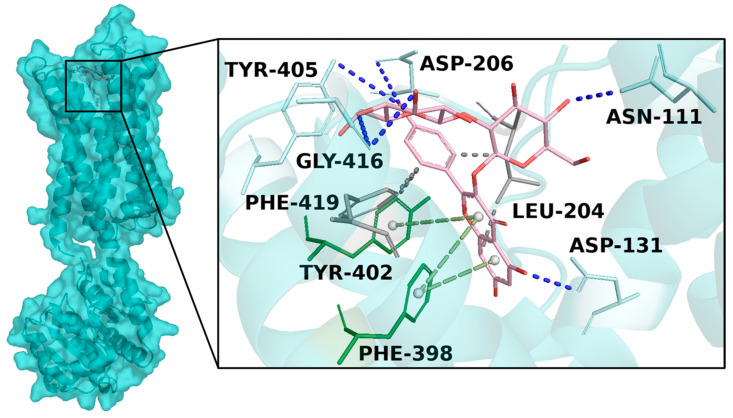
Overview of human adrenergic receptor Alpha-2 docked with KOS-3. The target 3D structure is depicted in cyan surface and cartoon, while KOS-3 is reported in pink (carbon atom), red (oxygen atom), and white (hydrogen atom) sticks. Enlarged view of the docked pose of KOS-3 within the target binding pocket. In gray, cyan and green sticks are represented the binding residue forming hydrophobic interactions (gray dotted line), h-bonds (blue dotted line), and π-stacking (green dotted line), respectively.

Interaction network analyses revealed a large hydrophobic and polar interaction network with sensing residues of the target; in detail, KOS-3 formed hydrophobic interactions with Leu-204, Tyr-402, and Phe-419; h-bonds with Asn-111, Asp-131, Asp-206, Tyr-405, and Gly-416; and two π-stacking with Phe-398 and Tyr-402 (Figure 5).

### 3.4. Mutagenicity Assay: Ames Test

**Figure 6:**
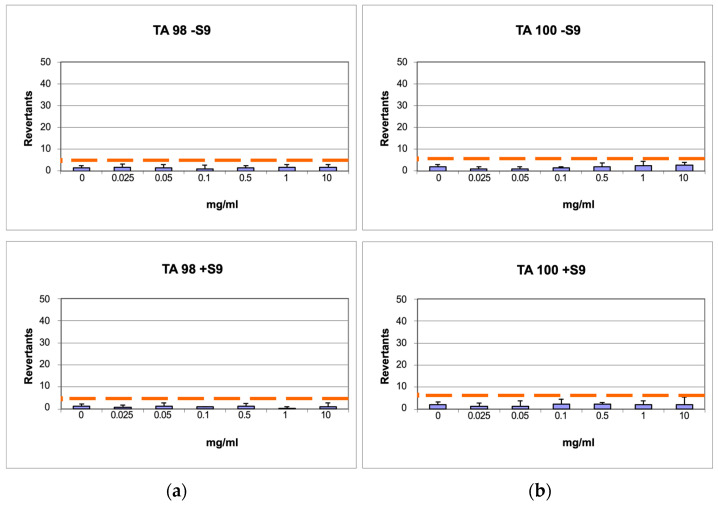
Number of revertants in TA98 (**a**) and TA100 (**b**) *S. typhimurium* strain treated with increasing concentrations of CST with and without S9 fraction. The results are reported as the mean of revertants ± SD; *n* = 6; *p* ≤ 0.01.

To rule out any possible mutagenic effect of CST, six concentrations of the extract were assessed on TA98 and TA100 bacteria in the *Salmonella* mutagenicity assay, both with and without S9 metabolic activation. The results in Figure 6a,b show that CST did not exhibit genotoxicity towards TA98 and TA100 at any concentration tested, whether S9 activation was present or not. Specifically, the number of revertants remained consistently lower and significantly different from the positive control up to a concentration of 10,000 µg/mL (*p* ≤ 0.01). In all instances, both the background and positive control levels were within the typical range observed in our laboratory.

### 3.5. LCA Analysis

The goal of the LCA study focused on the extraction process was the environmental footprint calculation of the extraction process of bioactive substances from saffron tepal biomass. The scope was to generate a specific LCA dataset useful for crediting the potential environmental benefits from replacing a traditional product on the market with the development of bio-based alternative.

In Table 4, we report the characterization results of the environmental footprint of 1 g of saffron bioactive substances extract.

In general, the drying step contributed mostly to the eco-profile, followed by the hydroalcoholic extraction and freeze-drying steps. The indicator values highlight that the “climate change” and “resource use, fossil” categories were mainly affected by electricity consumption, which, in general, determined most of the impact on all categories. This outcome was clearly affected by the assumptions taken in this study (electricity from the Italian energy mix, at medium voltage) that would need to be evaluated and replaced by measured data for specific use. Furthermore, solvent recovery/recycling (not considered in this analysis) could reduce the impact on the ecotoxicity category.

## 4. Discussion

The advantages of using plant-based metabolites, particularly those from waste biomasses, for potential therapeutic applications are increasingly recognized today. An integrated approach to repurposing plant waste offers significant advantages that enhance both sustainability and efficacy. By combining extraction techniques with phytochemical characterization, researchers can efficiently identify and extract valuable bioactive compounds. In vitro and in silico mechanistic studies provide insights into the biological activities of these compounds, highlighting their potential health benefits. Additionally, incorporating LCA studies allows for a thorough evaluation of the environmental impacts associated with extraction and utilization processes. This holistic approach maximizes the value of plant waste, supports sustainable practices, and contributes to the circular bioeconomy, ultimately transforming waste into valuable resources for pharmaceuticals, nutraceuticals, and functional foods. With this in mind, our study focused on the floral bio-residues of *C. sativus* as a valuable resource within the framework of a circular bioeconomy.

The first step in embracing sustainability was carefully selecting our starting material. Tepals were collected from a farm in Tuscany that utilizes permaculture practices. Permaculture is a design system often applied in the design of farms, or in agricultural initiatives. It was defined in 1978 in Australia by Bill Mollison and David Holmgren [85] and has gained worldwide attention. We can count more than 5000 projects that have been designed applying the permaculture process, and more than one million people that have been certified as designers. It is taught in universities in Australia, the USA, and the EU, with a pilot project in five universities in five different countries, including the University of Catania in Italy. The design system is based on three ethics: earth care, people care, and future care; and on a clear and defined process of design that uses principles and various strategies to analyze the potential of a project and the best layout it should have. Permaculture has a systems thinking approach to design that originates from the observation of natural ecosystems. It has a clear connection with the circular bioeconomy because it tries to work on energy efficiency and the reduction, or total exclusion, of waste. Working from an ecosystemic perspective, where in nature, waste does not exist, permaculture tries to find solutions to reduce completely the presence of waste in any project [86]. Applied in a farm setting, permaculture creates a system where farming is connected to the wider context. Farming becomes part of the ecosystem and is not an exploitative initiative, but a regenerative one. Growing saffron is interesting under a permaculture perspective because it is a self-reproducing bulb that gives the farm a never-ending supply of resources that will grow in time. The use of tepals, even though they are edible, is not common and often they are seen as waste. Reintroducing them in a production chain is a clear objective for a permaculture farm.

Used for medicinal purposes since ancient times, extensive phytochemical and biochemical studies have isolated numerous bioactive ingredients from saffron [25]. Key bioactive substances include crocin, crocetin, picrocrocin, safranal, and kaempferols [25,26,27,28,29,87]. Crocin, picrocrocin, and safranal (picrocrocin aglycone) are degradation products of carotenoids; known as apocarotenoids, they are responsible for saffron’s organoleptic properties and are mainly found in the stigma [19,22,23,24]. However, crocin is also present in the by-products of *C. sativus* [24]. Crocetin esters contribute to saffron’s red color, picrocrocin to its bitter taste, and safranal to its aroma [27]. Overall, saffron contains over 150 volatile and flavoring compounds, including terpenes and their esters, and several non-volatile compounds, including the carotenoids zeaxanthin, lycopene, α- and β-carotenes, which, together with crocins, contribute to its color. Kaempferols are noted for their antioxidant activity and are used in dietary supplements, functional foods, pharmaceutical preparations, and cosmetics [27,29,41,88]. The perianths of *C. sativus* flowers contain high levels of phenolics, primarily anthocyanins, and flavonols, with kaempferol glycosides representing 70–90% of the total flavonoid content [89,90]. While saffron stigmas contain only kaempferol glycosides as flavonoids, saffron tepals encompass a broader range of flavonoid compounds. These include anthocyanins, quercetin-related compounds, and flavones [91]. Studies on saffron petal extracts in mice demonstrated anti-nociceptive effects against chemically induced pain. The ethanolic extract reduced chronic inflammation, likely due to the presence of flavonoids, tannins, anthocyanins, alkaloids, and saponins [92]. The tepals of *C. sativus*, particularly, can yield high amounts of kaempferols, which are powerful anti-inflammatory agents [27].

Phenolic compounds and carotenoids in saffron protect against oxidative damage [47,48,93]. Flavonols and their glycosides are recognized for their antioxidant activity [27] and contribute to the health benefits traditionally attributed to saffron [29]. Extracting these secondary metabolites from waste by-products is crucial for repurposing waste material and obtaining high-quality products. The GRAS (Generically Recognized As Safe) [94] ethanol–water (70:30 *v*/*v*) mixture was chosen as the solvent for extraction due to its safety and handling properties. Conventional techniques for extracting flavonoids from plant matrices typically involve various solvent combinations such as water, ethanol, methanol, acetone, and ethyl acetate, either individually or in combination. These methods often yield high extraction efficiencies of compounds with antioxidant properties [95].

The TPC and TFC of the extract were measured to assess extraction efficiency. CST exhibited a TPC of 80.05 (±5.11) mg GAE/g dry extract, higher than the methanolic extract of Moroccan *C. sativus* tepals (65.34 ± 1.74 mg GAE/g dry extract) [26], and a TFC of 38.36 (±1.22) mg QE/g, even using milder “green” solvents, thereby promoting sustainability. The presence of phenolic compounds in the extract, including flavonoids, translated into notable RP (reducing power) and scavenging capabilities.

UPLC-MS/MS profiling confirmed the high abundance and variety of flavonoids in CST, including flavones, flavonols, and flavanones. KOS-3 was identified as the main component, and previous reports indicated it as the principal secondary metabolite in *C. sativus* flowers [90]. Studies have shown that kaempferol and its glycosides possess antioxidant and anti-inflammatory activities [29,41,88], and KOS-3 is also valued for these properties [90,96,97,98,99].

The second most abundant compound found in CST was Astragalin, or Kaempferol 3-O-glucoside. Studies have identified KOS-3 and Astragalin as important qualitative and quantitative marker compounds of saffron tepals [90]. Astragalin is found in various medicinal plants, showing diverse pharmacological properties (anti-inflammatory, antioxidant, neuro- and cardioprotective, anti-osteoporotic, antitumoral, and others [100]. It produces these effects by influencing a range of molecular targets, including transcription factors; a variety of enzymes; different types of kinases; cell adhesion proteins, both apoptotic and anti-apoptotic proteins; along with inflammatory cytokines [100,101,102].

In addition, 6-Hydroxyluteolin, a hydroxy-derivative of luteolin, was identified. Lim et al. reported that luteolin and genistein, another flavonoid identified in CST, effectively reduced elevated levels of IL-1β and IL-6 inflammatory cytokines and NF-κB activation in animal models [39]. Luteolin can contrast inflammation by reducing the production of inflammatory markers such as NO, prostaglandin E2 (PGE2) and iNOS and COX-2 precursor enzymes, TNF-α, and various matrix metalloproteinases (MMPs), as demonstrated in both in vitro and in vivo models of arthritis, indicating its potential as an effective anti-inflammatory agent [103].

Quercetin and derivatives were also found in a notable amount in CST. Previous research has investigated the effects of isorhamnetin, a 3′-O-methylated metabolite of quercetin, on various biological processes, including iNOS expression, NO release, LPS-induced ROS production, and apoptosis. Additionally, isorhamnetin was found to enhance the expression of heme oxygenase-1 (HO-1) by modulating the translocation of NF-E2-related factor-2 (Nrf2). Results suggested that its antioxidant properties play a fundamental role in its anti-inflammatory effects, particularly through the inhibition of COX-2 expression during inflammatory responses [104]. Moreover, numerous investigations on isorhamnetin have highlighted its wide-ranging benefits, including its ability to protect cardiovascular and cerebrovascular health, exert anti-tumor effects, reduce inflammation, provide antioxidant protection, safeguard organs, and prevent obesity. The underlying mechanisms through which isorhamnetin operates involve the regulation of several critical signaling pathways, such as PI3K/AKT/PKB, NF-κB, and MAPK. Additionally, it influences the expression and activity of various cytokines and kinases that play roles in these protective and therapeutic effects [105].

We used different cell models to evaluate the potential use of CST for diverse applications. The ability to modulate H_2_O_2_-induced oxidative stress and the fermentation capabilities in yeast cells was evaluated using the *S. cerevisiae* K310 strain. *S. cerevisiae* is the most extensively studied eukaryote system, serving as a valuable model organism for basic research due to its unicellular nature and the conservation of eukaryotic biological functions [106]. Its ease of genetic manipulation and significant biotechnological applications, particularly in fermentation, alcohol, and CO_2_ production, and its ability to thrive in challenging environmental conditions, further enhance its importance. Key applications of *S. cerevisiae* include, but are not limited to, its use in the food and beverage industries, particularly in wine production, as well as its growing role in industrial applications for biofuel production [106,107,108]. Innovations in biotechnology are driving advancements in the large-scale production of biofuels, such as bioethanol, a clean renewable energy source, through the use of model microorganisms in bio-fermentation [107]. Alcohol fermentation is a critical step in these processes, and improving fermentation efficiency is essential for both sectors. This study demonstrated that treating *S. cerevisiae* with CST enhanced ethanol yields, even under oxidative stress conditions. This improvement was attributed to CST’s ability to scavenge oxidative molecules, as evidenced by radical scavenging tests and in vitro viability assays on *S. cerevisiae*. Moreover, in silico analyses showed the ability of KOS-3 to bind in a sensing region of yeast AR, triggering a wide polar interaction network with important residues of the target This suggests that the compound has the potential to influence the biological activity of the target. Yeast AR is encoded by the GRE3 gene, which is upregulated under various stress conditions, including NaCl and H_2_O_2_ [109]. AR enzyme plays a significant role in the polyol pathway, which is involved in the metabolism of glucose. Under normal physiological conditions, AR converts glucose to sorbitol using NADPH as a cofactor. However, under non-physiological conditions, this pathway becomes overactive, leading to an accumulation of sorbitol and subsequent oxidative stress [110]. Studies have shown that the deletion of the GRE3 gene in engineered *S. cerevisiae* significantly reduces the accumulation of undesirable byproducts, such as xylitol, thereby enhancing yeast fermentation efficiency [111]. Other studies have also reported the ability of plant extracts to enhance ethanol fermentation efficiency in *S. cerevisiae* through their antioxidant properties [108]. This study is the first to report that *C. sativus* tepals extract reduces oxidative stress and enhances ethanol fermentation in *S. cerevisiae*. Along with uncovering new molecular mechanisms behind the antioxidant effects of the extract, this research could pave the way for intriguing new applications in the food and biofuels sectors.

The antioxidant and anti-inflammatory effects of CST were further validated using RAW 264.7 macrophages. Treatment with CST resulted in a concentration-dependent decrease in LPS-mediated production of ROS. Additionally, CST effectively reduced the secretion of NO and lowered the expression of the enzymes iNOS and COX-2 in stimulated RAW 264.7 cells. It also inhibited the phosphorylation of ERK, JNK, and p38 MAPKs. Analysis of the nuclear expression of the p65 subunit of the NF-κB transcription factor, along with immunofluorescence studies, demonstrated that pretreatment with CST at the highest concentration led to the retention of NF-κB p65 in the nucleus following LPS stimulation. Target prediction and docking simulations provided new insights into the mechanisms behind the effects of *C. sativus* tepals extracts. Specifically, we found that KOS-3 has a strong binding affinity for the adrenergic receptor Alpha-2 in a region associated with interactions with known active compounds [112].

Adrenoceptors are a type of G protein-coupled receptor that primarily interact with catecholamines like norepinephrine and epinephrine. These receptors are found throughout various cell types in the body. When catecholamines bind to adrenoceptors, they activate the sympathetic nervous system, influencing blood pressure, heart muscle contraction, and several central nervous system functions. Adrenergic agents, including both agonists and antagonists, have a broad range of therapeutic uses. They are effective in treating a variety of conditions, including cardiovascular diseases, asthma, depression, oxidative stress, and inflammatory disorders, among others [113,114]. Oxidative stress refers to the damage caused to tissues, cells, and biological macromolecules by excess oxidizing agents, resulting in metabolic changes, DNA and protein damage, and cell death [115]. While the body possesses endogenous antioxidant systems, it also depends on exogenous antioxidants obtained from the diet [116]. *C. sativus* flowers are particularly abundant in these antioxidant compounds, which can counteract free radicals and enhance the body’s defenses against oxidative stress by activating relevant pathways [27]. The interplay between ROS and inflammatory markers, including NO, iNOS, and COX-2, is crucial for regulating inflammation and maintaining homeostasis [117]. The production of ROS can activate iNOS, leading to increased NO levels, which can further exacerbate oxidative stress and inflammation. Additionally, the interaction between ROS and NO can trigger the NF-κB and MAPK pathways, promoting the expression of pro-inflammatory cytokines and other inflammatory mediators [118]. This cascade also enhances the production of COX enzymes, particularly COX-2, which are responsible for synthesizing prostaglandins that mediate inflammatory responses, contributing to various pathological conditions [119]. Adrenergic receptor Alpha-2 activates MAPK and Akt upon interaction with norepinephrine [120]. Interestingly, our simulation provided evidence that KOS-3 bound to a different region of the adrenergic receptor Alpha-2, triggering a distinct interaction network [121]; such evidence would suggest that our compound may exert antioxidant activity by binding to a known antioxidant active site of the target [112]. Additionally, it may act as an anti-inflammatory agent by blocking MAPK activation. Furthermore, the high homology between human and mouse ADRA2C and the full conservation of the consensus binding residues identified in the docking simulation suggest a similar binding mode and potential biological activity of KOS-3 against the mouse target, strongly supporting the in vitro evidence.

These findings not only support the extract’s ability to address critical aspects of inflammation but also highlight novel mechanisms that could assist in creating biotechnological products focused on alleviating inflammation, with potential uses in pharmaceuticals, cosmeceuticals, and nutraceuticals. Indeed, cosmetics utilize *C. sativus’* anti-aging and anti-inflammatory effects, contributing to skin health and beauty products [42,122,123,124]. Petal extracts are increasingly used in high-end cosmetics to combat aging by lowering advanced glycation end products (AGEs)—harmful compounds formed when proteins or lipids become glycated, which exacerbate oxidative stress and inflammation [27]. Crocin has been shown to reduce UVB-induced damage by decreasing ROS levels, making it effective in preventing photoaging [87]. Additionally, hydrogel enriched with antioxidant compounds from saffron petals has proven beneficial for difficult wound care, such as burns and ulcers, and can also be used for sensitive skin treatments [125]. Current data suggest that *C. sativus* flower extracts contain phytochemicals that promote wound healing and increase VEGF levels during skin repair, although further studies are needed to identify the specific compounds responsible for this effect [126]. Based on our results from the Ames test, which evaluates the potential of a chemical to induce DNA mutations in bacteria, we found no evidence of mutagenicity in CST. Since none of the tested concentrations exceeded the threshold limit associated with reversion due to potential mutations in the histidine biosynthetic pathway enzyme, we can conclude that CST can be safely utilized within certain limits in the pharmaceutical, nutraceutical, and cosmetic industries.

Based on our findings and the existing literature, this study emphasizes the potential of utilizing waste-derived extracts through “green” methodologies in various biotechnological applications, supported by both bioactivity studies and LCA. We employed in vitro assays and in silico analyses to evaluate the anti-inflammatory and antioxidant properties of the extract, reflecting our commitment to ethical research practices. By focusing on these methods, we adhered to the principles of the 3Rs (Replacement, Reduction, and Refinement) [127], which emphasize minimizing the use of animal models. This approach allowed us to gather essential data while reducing the need for animal testing at this stage. When integrated with LCA studies, it offers opportunities to align health innovations with the principles of a circular bioeconomy, paving the way for a more sustainable and health-conscious future. The LCA analysis highlights the environmental benefits of recovering *C. sativus* waste to eco-design new potential value chains to replace traditional products on the market while providing a valuable dataset for future assessments. This perspective enhances the importance of the present work by promoting an integrated approach that combines in silico and in vitro studies with the added value of LCA for waste repurposing, rather than merely focusing on extraction and the biological activity of the extract. Considering the numerous applications of *C. sativus* by-products [15,23,125,128,129] and plant waste in general [3,6,14,130,131,132,133], which span a wide range of biotechnological applications across various industries, this approach could lead to the development of a comprehensive workflow model. Such a model may provide significant advantages for further evaluations compared to traditional methodologies.

## 5. Conclusions

The current study provides strong evidence that repurposing *C. sativus* waste, particularly the tepals, can significantly support the circular bioeconomy by recovering valuable bioactive compounds. We demonstrated the antioxidant and anti-inflammatory properties of a hydro-ethanolic extract from saffron tepals sourced in Tuscany, Italy. Comprehensive phytochemical analysis revealed potent antioxidant compounds, validated through various spectrophotometric and biological assays. The extract’s antioxidant capabilities were confirmed in the model organism *S. cerevisiae*, where it mitigated oxidative stress and enhanced fermentative capacity. Additionally, the extract showed significant anti-inflammatory effects in LPS-stimulated RAW 264.7 cells, indicating potential therapeutic applications. In silico analysis provided insights into the mechanisms behind these effects. The Life Cycle Assessment highlighted the environmental benefits of utilizing saffron waste. This research underscores the value of saffron tepals for the pharmaceutical, nutraceutical, and cosmetic industries while advocating for sustainable practices within the bioeconomy framework.

## Figures and Tables

**Table 1:** Target details.

Target	Common Name	UniProt ID	Target Class	Probability Score (%)
Neuromedin-U receptor 2	NMUR2	P48645	Family A G protein-coupled receptor	73
Alpha-2a adrenergic receptor	ADRA2A	P08913	Family A G protein-coupled receptor	73
adrenergic receptor Alpha-2	ADRA2C	P18825	Family A G protein-coupled receptor	73
Acetylcholinesterase	ACHE	P22303	Hydrolase	73
Aldose reductase	AKR1B1	P15121	Enzyme	39

**Table 2:** TPC, TFC, and antioxidant capacity of CST.

			Antioxidant Capacity		
	TPC (mg GAE/g)	TFC (mg QE/g)	RP (mg AAE/g)	ABTS (IC50 µg/mL)	DPPH (IC50 µg/mL)
CST	80.05 (±5.11)	38.36 (±1.22)	52.89 (±2.13)	113.07 (±2.37)	374.22 (±20.83)

Note: *TPC*, total phenolic content; *TFC*, total flavonoid content; *TRP*, total reducing power; *ABTS*, 2,2′-azino-bis(3-ethylbenzothiazoline-6-sulfonic acid); *DPPH*, 2,2-diphenyl-1-picrylhydrazyl; *GAE*, gallic acid equivalent; *QE*, quercetin equivalent; *AAE*, ascorbic acid equivalent. Data are expressed as mean (±SD), *n* = 3.

**Table 3:** Matched metabolites in CST ethanolic extract.

Name	Retention Time (min)	Formula	Calculated MW	*m*/*z*	Reference Ion	Mass Error (ppm)	Peak Area (%)
Kaempferol 3-O-sophoroside	14.999	C_27_H_30_O_16_	610.15712	609.1498	[M-H]^−1^	6.12	51.95
Astragalin	17.129	C_21_H_20_O_11_	448.10402	449.1095	[M+H]^+1^	3.51	12.50
Kaempferol	23.266	C_15_H_10_O_6_	286.04725	285.04	[M-H]^−1^	−1.69	10.56
6-Hydroxyluteolin	14.266	C_15_H_10_O_7_	302.04433	303.0516	[M+H]^+1^	4.94	9.32
Isorhamnetin 3,4′-diglucoside	10.587	C_28_H_32_O_17_	640.16692	641.1742	[M+H]^+1^	4.64	3.03
Adenosine	3.684	C_10_H_13_N_5_O_4_	267.09639	268.1037	[M+H]^+1^	−1.35	2.54
Isorhamnetin 3-O-robinobioside	16.094	C_28_H_32_O_16_	624.1731	623.166	[M-H]^−1^	6.52	2.10
Quercetin-3-O-glucoside	14.165	C_21_H_20_O_12_	464.09761	465.1049	[M+H]^+1^	4.6	1.90
Kaempferide	2.033	C_16_H_12_O_6_	300.0603	299.053	[M-H]^−1^	6.25	1.08
Apigenin 7-sophoroside	15.922	C_27_H_30_O_15_	594.16279	593.1561	[M-H]^−1^	7.27	0.90
Daidzein	26.625	C_15_H_10_O_4_	254.05749	253.0502	[M-H]^−1^	−1.64	0.74
9,10-Dihydro-3,8-dihydroxy-1-methyl-9,10-dioxo-2-anthracenecarboxylic acid	26.631	C_16_H_10_O_6_	298.04756	297.0403	[M-H]^−1^	−0.6	0.59
Crocin 3	23.189	C_32_H_44_O_14_	652.27507	675.2643	[M+Na]^+1^	3.02	0.51
Quercetin 3-O-gentiobioside	5.276	C_27_H_30_O_17_	626.14799	627.1552	[M+H]^+1^	−0.5	0.36
Quercetin	20,816	C_15_H_10_O_7_	302.04262	301.0353	[M-H]^−1^	−0.12	0.30
Kaempferol 3,7,4′-triglucoside	15.072	C_33_H_40_O_21_	772.20758	773.2148	[M+H]^+1^	1.78	0.27
Genistein	16.658	C_15_H_10_O_5_	270.05428	271.0616	[M+H]^+1^	5.38	0.21
Perlolyrin	17.105	C_16_H_12_N_2_O_2_	264.09032	265.0976	[M+H]^+1^	1.67	0.20
Myricetin	18.278	C_15_H_10_O_8_	318.04039	317.0331	[M-H]^−1^	8.87	0.17
Safranal	24.766	C_10_H_14_O	150.10477	151.1121	[M+H]^+1^	2.02	0.11
Apigenin	16.509	C_15_H_10_O_5_	270.05449	271.0618	[M+H]^+1^	6.18	0.11
3-Hydroxy-beta-ionone	29.51	C_13_H_20_O_2_	208.14577	207.1385	[M-H]^−1^	−2.71	0.10
Eriodictyol	18.462	C_15_H_12_O_6_	288.06355	287.0563	[M-H]^−1^	0.57	0.10

**Table 4:** Environmental footprint characterization results for 1 g of CST.

Indicators	Impact Result	Unit
Acidification	0.005	mol H+ eq
Climate change	2.360	kg CO_2_ eq
Ecotoxicity, freshwater	0.096	CTUe
Eutrophication marine	0.001	kg N eq
Eutrophication, freshwater	1.85 × 10^−5^	kg N eq
Eutrophication, terrestrial	0.012	mol N eq
Human toxicity, cancer	2.70 × 10^−9^	CTUh
Human toxicity, non-cancer	2.66 × 10^−8^	CTUh
Ionising radiation, human health	0.439	kBq U-235 eq
Land use	24.579	Pt
Ozone depletion	7.79 × 10^−10^	kg CFC11 eq
Particulate Matter	4.00 × 10^−8^	disease inc.
Photochemical ozone formation—human health	0.026	kg NMVOC
Resource use, fossils	34.952	MJ
Resource use, minerals and metals	1.14 × 10^−6^	kg Sb eq
Water use	0.589	m^3^ depriv.

## Data Availability

The original contributions presented in the study are included in the article/Supplementary Material; further inquiries can be directed to the corresponding author.

## Supplementary Materials

The following supporting information can be downloaded at: https://www.mdpi.com/article/10.3390/antiox13091082/s1, Table S1: Complete list of metabolites found in CST. Figure S1: System boundaries of the extraction process of bioactive substances from saffron petal biomass. Figure S2: Pairwise sequence alignment between human and mouse ADRA2C.
