# Supplementary material for: Exploring the Antioxidant and Anti-Inflammatory Potential of Saffron (Crocus sativus) Tepals Extract within the Circular Bioeconomy
Source: Antioxidants (Basel). 2024 Sep 4;13(9):1082. doi: 10.3390/antiox13091082 (PMC11428576; doi:10.3390/antiox13091082)
Supplement: Supplementary file 1 [file antioxidants-13-01082-s001.zip › Figure S2.pdf]

```
Identities:416/452(92%), Positives:419/452(92%), Gaps:4/452(0%)

Human  1    MASPALAAALAVAAAAGPNASGAGERGSGGVANASGASWGPPRGQYSAGAVAGLAAVVGF  60
            MASPALAAALA AAA GPN S AGE GSGG ANASG  W PP GQYSAGAVAGLAAVVGF
Mouse  1    MASPALAAALAAAAAEGPNGSDAGEWGSGGGANASGTDWVPPPGQYSAGAVAGLAAVVGF  60

Human  61   LIVFTVVGNVLVVIAVLTSRALRAPQNLFLVSLASADILVATLVMPFSLANELMAYWYFG  120
            LIVFTVVGNVLVVIAVLTSRALRAPQNLFLVSLASADILVATLVMPFSLANELMAYWYFG
Mouse  61   LIVFTVVGNVLVVIAVLTSRALRAPQNLFLVSLASADILVATLVMPFSLANELMAYWYFG  120

Human  121  QVWCGVYLALDVLFCTSSIVHLCAISLDRYWSVTQAVEYNLKRTPRRVKATIVAVWLISA  180
            QVWCGVYLALDVLFCTSSIVHLCAISLDRYWSVTQAVEYNLKRTPRRVKATIVAVWLISA
Mouse  121  QVWCGVYLALDVLFCTSSIVHLCAISLDRYWSVTQAVEYNLKRTPRRVKATIVAVWLISA  180

Human  181  VISFPPLVSLYRQPDGAAYPQCGLNDETWYILSSCIGSFFAPCLIMGLVYARIYRVAKLR  240
            VISFPPLVS YR+PDGAAYPQCGLNDETWYILSSCIGSFFAPCLIMGLVYARIYRVAKLR
Mouse  181  VISFPPLVSFYRRPDGAAYPQCGLNDETWYILSSCIGSFFAPCLIMGLVYARIYRVAKLR  240

Human  241  TRTLSEKRAPVGPDGASPTTENGLGAAAGAGENGHCAPPPADVEPDESSAAAERRRRRGA  300
            TRTLSEKR P GPDGASPTTENGLG A  AGENGHCAPP  +VEPDESSAA  RRRR  A
Mouse  241  TRTLSEKRGPAGPDGASPTTENGLGKA--AGENGHCAPPRTEVEPDESSAAERRRRRG-A  297

Human  301  LRRGGRRRAGAEGGAGGADGQGAGPGAAESGALTASRSPGPGGRLSRASSRSVEFFLSRR  360
            LRRGGRRR GAEG  G ADG G G  AAE GA TASRSPGPGGRLSRASSRSVEFFLSRR
Mouse  298  LRRGGRRREGAEGDTGSADGPGPG-LAAEQGARTASRSPGPGGRLSRASSRSVEFFLSRR  356

Human  361  RRARSSVCRRKVAQAREKRFTFVLAVVMGVFVLCWFPFFFSYSLYGICREACQVPGPLFK  420
            RRARSSVCRRKVAQAREKRFTFVLAVVMGVFVLCWFPFFFSYSLYGICREACQ+P PLFK
Mouse  357  RRARSSVCRRKVAQAREKRFTFVLAVVMGVFVLCWFPFFFSYSLYGICREACQLPEPLFK  416

Human  421  FFFWIGYCNSSLNPVIYTVFNQDFRRSFKHIL  452
            FFFWIGYCNSSLNPVIYTVFNQDFRRSFKHIL
Mouse  417  FFFWIGYCNSSLNPVIYTVFNQDFRRSFKHIL  448
```

Figure S2. Pairwise Sequence Alignment (PSA). PSA between human (first row) and mouse (second row) ADRA2C. The central row reports the conservation of the residues along the sequence alignment. When the residue is conserved, in the central row is reported the corresponding letter of the residue, while the signs "+", "-", and " " represent positives, gap, and no conservation (along the sequence alignment), respectively. The residues enclose in the red box represent the *consensus* binding residues.
