# Supplementary material for: Exploring the Antioxidant and Anti-Inflammatory Potential of Saffron (Crocus sativus) Tepals Extract within the Circular Bioeconomy
Source: Antioxidants (Basel). 2024 Sep 4;13(9):1082. doi: 10.3390/antiox13091082 (PMC11428576; doi:10.3390/antiox13091082)
Supplement: Supplementary file 1 [file antioxidants-13-01082-s001.zip › Table S1.pdf]

Table S1. Complete list of metabolites found in CST.

Name	Retention time (min)	Formula	Calculated MW	m/z	Reference ion	Mass error (ppm)	Peak Area (%)
Kaempferol 3-O-sophoroside	14.999	C27H30O16	610.15712	609.14984	[M-H]-1	6.12	51.95
Astragalin	17.129	C21H20O11	448.10402	449.1095	[M+H]+1	3.51	12.50
Kaempferol	23.266	C15H10O6	286.04725	285.03998	[M-H]-1	-1.69	10.56
6-Hydroxyluteolin	14.266	C15H10O7	302.04433	303.05161	[M+H]+1	4.94	9.32
Isorhamnetin 3,4'-diglucoside	10.587	C28H32O17	640.16692	641.17419	[M+H]+1	4.64	3.03
Adenosine	3.684	C10H13N5O4	267.09639	268.10367	[M+H]+1	-1.35	2.54
Isorhamnetin 3-O-robinobioside	16.094	C28H32O16	624.1731	623.16595	[M-H]-1	6.52	2.10
Quercetin-3-O-glucoside	14.165	C21H20O12	464.09761	465.10489	[M+H]+1	4.6	1.90
Kaempferide	2.033	C16H12O6	300.0603	299.05302	[M-H]-1	6.25	1.08
Apigenin 7-sophoroside	15.922	C27H30O15	594.16279	593.15607	[M-H]-1	7.27	0.90
Daidzein	26.625	C15H10O4	254.05749	253.05022	[M-H]-1	-1.64	0.74
9,10-Dihydro-3,8-dihydroxy-1-methyl-9,10-dioxo-2-anthracenecarboxylic acid	26.631	C16H10O6	298.04756	297.04028	[M-H]-1	-0.6	0.59
Crocin 3	23.189	C32H44O14	652.27507	675.26428	[M+Na]+1	3.02	0.51
Quercetin 3-O-gentiobioside	5.276	C27H30O17	626.14799	627.15521	[M+H]+1	-0.5	0.36
Quercetin	20.816	C15H10O7	302.04262	301.03534	[M-H]-1	-0.12	0.30
Kaempferol 3,7,4'-triglucoside	15.072	C33H40O21	772.20758	773.21484	[M+H]+1	1.78	0.27
Genistein	16.658	C15H10O5	270.05428	271.06155	[M+H]+1	5.38	0.21
Perlolyrin	17.105	C16H12N2O2	264.09032	265.0976	[M+H]+1	1.67	0.20
Myricetin	18.278	C15H10O8	318.04039	317.03311	[M-H]-1	8.87	0.17
Safranal	24.766	C10H14O	150.10477	151.11205	[M+H]+1	2.02	0.11
Apigenin	16.509	C15H10O5	270.05449	271.06177	[M+H]+1	6.18	0.11
3-Hydroxy-beta-ionone	29.51	C13H20O2	208.14577	207.13849	[M-H]-1	-2.71	0.10
Eriodictyol	18.462	C15H12O6	288.06355	287.05627	[M-H]-1	0.57	0.10
Quercetin 3- (2"-galoylrutinoside)	12.001	C34H34O20	762.16557	763.17285	[M+H]+1	1.62	tr.
Rhamnalpinogenin	17.091	C17H12O7	328.05861	329.06589	[M+H]+1	0.94	tr.
EPIGALLOCATECHIN 3-O-(3-O-METHYL)GALLATE	15.596	C23H20O11	472.10143	473.1087	[M+H]+1	1.83	tr.
Naringin	17.505	C27H32O14	580.18458	579.17731	[M-H]-1	9.27	tr.
Epigallocatechin-(4beta->8)-epicatechin-3-O-gallate ester	10.72	C37H30O17	746.14998	745.1427	[M-H]-1	2.25	tr.
Quercetin 3-sophorotrioside	12.375	C33H40O22	788.20252	787.19524	[M-H]-1	1.77	tr.
FLAVANONE	25.795	C15H12O2	224.08351	223.07623	[M-H]-1	-0.98	tr.
Quercetin 3-rutinoside-7-glucuronide	12.723	C33H38O22	786.18782	785.18054	[M-H]-1	2.98	tr.
QUERCETIN 3,7-DIGLUCOSIDE	17.054	C27H30O17	626.14952	627.1568	[M+H]+1	1.95	tr.
Crocetin glucosyl ester	24.752	C26H34O9	490.22023	491.22751	[M+H]+1	-0.1	tr.
Kaempferol 3-sophorotrioside-7-glucoside	10.721	C39H50O26	934.26161	933.25433	[M-H]-1	2.76	tr.
Crocusatin H	24.561	C12H20O4	228.13594	227.12866	[M-H]-1	-0.97	tr.
quercetin 3-O-alpha-L-[6'''-p-coumaroyl-beta-D-glucopyranosyl-(1->2)-rhamnopyranoside]-7-O-beta-D-glucopyranoside	18.296	C42H46O23	918.24291	919.25018	[M+H]+1	-0.09	tr.
3-Formyl-6-hydroxy-2,4,4-trimethyl-2,5-cyclohexadien-1-one	22.942	C10H12O3	180.07873	181.086	[M+H]+1	0.45	tr.
Quercetin 3-rutinoside-7,3'-diglucoside	11.639	C39H50O26	934.26103	935.26831	[M+H]+1	2.14	tr.
Quercetin 3-O-(2G-Î²-D-xylopyranosylrutinoside)	14.542	C32H38O20	742.19518	743.20246	[M+H]+1	-0.62	tr.
Rutin	20.223	C27H30O16	610.1538	611.16108	[M+H]+1	0.68	tr.
Taxifolin	18.056	C15H12O7	304.05903	303.05176	[M-H]-1	2.41	tr.
Crocetin	27.012	C20H24O4	328.16707	329.17435	[M+H]+1	-1.18	tr.

Scutellarein 7-O-beta-glucopyranoside	13.128	C21H20O11	448.10136	447.09409	[M-H]-1	1.79	tr.
Chlorogenic acid	12.947	C16H18O9	354.09612	355.10339	[M+H]+1	2.92	tr.
Syringic acid	11.065	C9H10O5	198.0531	199.06038	[M+H]+1	1.41	tr.
Crocusatin B	30.454	C10H16O3	184.10974	185.11702	[M+H]+1	-1.09	tr.
Kaempferol 3,4',7-triacetate	19.909	C21H16O9	412.08003	413.08731	[M+H]+1	1.46	tr.
Crocusatin A	30.487	C9H14O2	154.09975	155.10703	[M+H]+1	2.39	tr.
(E)-p-coumaric acid	11.776	C9H8O3	164.04707	163.03979	[M-H]-1	-1.66	tr.
narcissin	19.622	C28H32O16	624.17044	625.17772	[M+H]+1	2.26	tr.
Crocin	23.82	C44H64O24	976.3803	975.37302	[M-H]-1	1.58	tr.
isorhamnetin 3-glucoside	18.716	C22H22O12	478.11241	477.10513	[M-H]-1	2.69	tr.
crocin 2	24.737	C38H54O19	814.32759	813.32031	[M-H]-1	2.04	tr.
Apigenin 6-C-glucosyl-7-O- (6-malyl-glucoside)	16.636	C31H34O19	710.17088	711.17816	[M+H]+1	2.05	tr.
quercetin 3-O-alpha-(6'''-caffeoylglucosyl-beta-1,2-rhamnoside)	18.646	C36H36O19	772.18486	773.19214	[M+H]+1	-0.28	tr.
Crocusatin F	18.552	C10H14O4	198.08874	197.08147	[M-H]-1	-2.35	tr.
Epijasminoside A	21.332	C16H26O7	330.16875	329.16147	[M-H]-1	2.71	100

tr. = traces (Area<0.1%)
