# Supplementary figures and images for: Exploring the Antioxidant and Anti-Inflammatory Potential of Saffron (Crocus sativus) Tepals Extract within the Circular Bioeconomy
Source: Antioxidants (Basel). 2024 Sep 4;13(9):1082. doi: 10.3390/antiox13091082 (PMC11428576; doi:10.3390/antiox13091082)

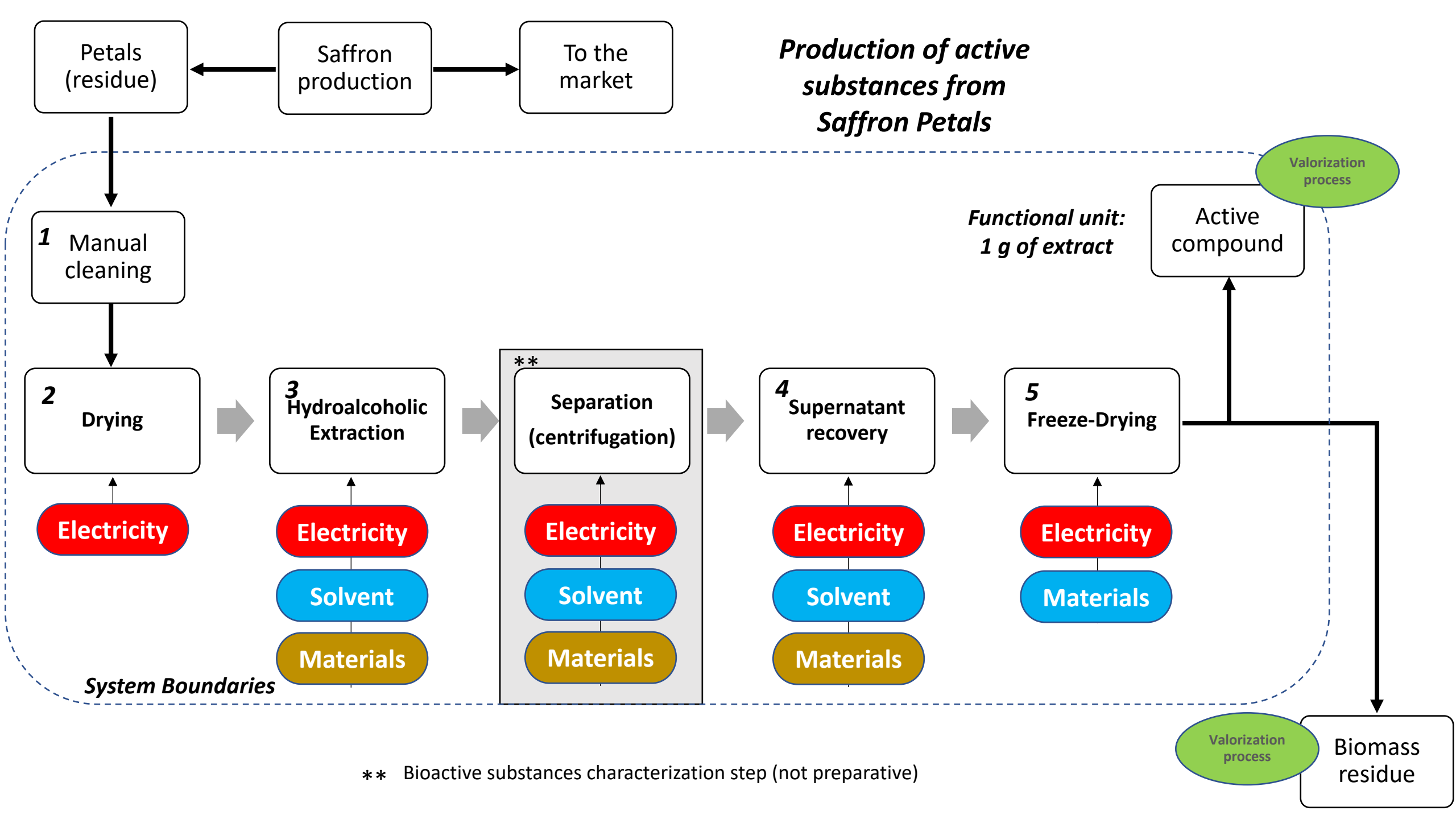

Supplement: Supplementary file 1 [file antioxidants-13-01082-s001.zip › Figure S1.pdf]
